# Revealing higher than expected meiofaunal diversity in Antarctic sediments: a metabarcoding approach

**DOI:** 10.1038/s41598-017-06687-x

**Published:** 2017-07-21

**Authors:** V. G. Fonseca, F. Sinniger, J. M. Gaspar, C. Quince, S. Creer, Deborah M. Power, Lloyd S. Peck, Melody S. Clark

**Affiliations:** 1 Zoological Research Museum Alexander Koenig (ZFMK), Centre for Molecular Biodiversity Research, Bonn, Germany; 2 Sesoko Station, Tropical Biosphere Research Center, University of the Ryukyus, 3422 Sesoko, Motobu, Okinawa, 905-0227 Japan; 3 Computational Biology Institute, George Washington University, Ashburn, Virginia USA; 4 Department of Microbiology and Infection, Warwick Medical School, University of Warwick, Coventry, CV4 7AL UK; 5 Molecular Ecology and Fisheries Genetics Laboratory, School of Biological Sciences, Bangor University, Gwynedd, LL57 2UW UK; 6 Centro de Ciencias do Mar, Universidade do Algarve, Campus de Gambelas, Faro, 8005-139 Portugal; 7 British Antarctic Survey, Natural Environment Research Council, High Cross, Madingley Road, Cambridge, CB3 0ET UK

## Abstract

An increasing number of studies are showing that Antarctic mega- and macrofauna are highly diverse, however, little is known about meiofaunal biodiversity in sediment communities, which are a vital part of a healthy and functional ecosystem. This is the first study to analyse community DNA (targeting meiofauna) using metabarcoding to investigate biodiversity levels in sediment communities of the Antarctic Peninsula. The results show that almost all of the meiofaunal biodiversity in the benthic habitat has yet to be characterised, levels of biodiversity were higher than expected and similar to temperate regions, albeit with the existence of potentially new and locally adapted species never described before at the molecular level. The Rothera meiofaunal sample sites showed four dominant eukaryotic groups, the nematodes, arthropods, platyhelminthes, and the annelids; some of which could comprise species complexes. Comparisons with deep-sea data from the same region suggest little exchange of Operational Taxonomic Units (OTUs) between depths with the nematodes prevalent at all depths, but sharing the shallow water benthos with the copepods. This study provides a preliminary analysis of benthic Antarctic Peninsula meiofauna using high throughput sequencing which substantiates how little is known on the biodiversity of one of the most diverse, yet underexplored communities of the Antarctic: the benthos.

## Introduction

Much recent effort has been expended into characterising Antarctic marine biodiversity and it is clear that it is significantly higher than was thought in previous decades, particularly in relation to marine invertebrates^1, 2^. An increasing number of cryptic species are being discovered^3^ and in some invertebrate groups, such as pycnogonids and polychaete worms, Antarctica has significantly higher diversity than the global averages^4^. However, even the most recent reviews of marine biodiversity in Antarctica have concentrated on marine mega- and macrofauna with relatively little discussion on endemic meiofauna, particularly those metazoans inhabiting marine sediments^5^. In a recent UN Assessment of the State of the Ocean I (http://www.worldoceanassessment.org/), meiofauna and the poles were highlighted as being of particular importance for future research. There is currently very limited knowledge on polar meiofauna; the extent of their biodiversity and their contribution to polar ecosystem functioning

Marine sediments are some of the most species-rich habitats on Earth. They are one of the main contributors to ocean health and functioning, but one of the least studied habitats in the biosphere^6^. Within marine sediments, the meiofauna (the microscopic taxa generally between 45–500 μm) are important members of the benthic ecosystem, playing a critical role in carbon transfer and nutrient cycling^6^. They participate in ecosystem energy flows via the consumption of dissolved organic carbon and from grazing on primary producers and bacteria^7^. In addition they play important roles in the consumption of detritus and predation. They excrete nutrients which can be used by phytobionts, bacteria and associated meiofauna, but they also act as a food source for benthic invertebrates and higher predators^6^. Thus, evaluations of benthic meiofauna biodiversity are of critical importance for understanding ecosystem functioning, sustainability and resilience, as well as understanding carbon cycling in the largest part of the World, the seabed^6^. In addition meiofauna represent useful tools for studying change within an ecosystem and could be particularly useful for understanding the effects of anthropogenic impacts and climate change^6^.

One region of particular note with respect to environmental change is the Western Antarctic Peninsula, some areas of which, particularly in the north west, are regarded as experiencing the most rapid rate of climate warming on the Antarctic continent^8^. However, the situation is complex and exacerbated by the lack of high density measurements. Recent analyses suggest that the atmospheric warming along the Peninsula has ceased^9^, but there is uncertainty whether this trend will continue, what the drivers are, and whether this cessation of warming is reflected in oceanographic data which is still showing changes in sea ice and retreat of glaciers^10^. What is clear is that this is still a region in transition and highly vulnerable^11^. Surface ocean temperatures rose by more than 1 °C in the second half of the 20^th^ Century and the deeper layers have also warmed due to increased upwelling of warm Upper Circumpolar Deep Water. Sea ice duration has reduced significantly in the past few decades (by 100 days since 1978), which impacts not only on primary production and water column stratification, but also on the frequency of iceberg scouring^11, 12^. About 80% of glaciers along the Peninsula are in retreat, which has increased the amount of sediment and fresh-water in the system^10^. Given the huge uncertainty concerning climate trends in this region, continued monitoring is vital, as is the evaluation of the potential impact on the endemic fauna. The Southern Ocean fauna have evolved to life in freezing seas in relative isolation for the last 15Myr^13^ and as a consequence have evolved a series of physiological and biochemical adaptations to life in the cold, are highly stenothermal and poorly adapted to rapid change^14^.

Advances in molecular and sequencing methodologies now enable us to evaluate biodiversity levels from even the most remote habitats, in a way, not previously possible. Large-scale environmental DNA (eDNA) approaches using high throughput sequencing (shortly referred to as metabarcoding) have recently been applied to examine biodiversity levels at the poles. To date polar marker gene studies have mainly focussed on microbial communities within soil, ice cores, microbial mats and melt water^15, 16^, marine viruses^17^, freshwater picoplankton^18^ and more recently, microbial biodiversity, on the shelf and the deep-sea^19, 20^. These studies have provided intriguing pilot data on micro- and meiofaunal biodiversity in this largely understudied and extreme environment. Whilst there is a long history of biological sediment analyses at research stations along the Peninsula, these have been based on either taxonomic identification or stable isotope analyses^21–25^. High throughput sequencing of DNA derived from community environmental samples provides a powerful tool with which to complement existing approaches and provides a timely opportunity to gain insight into alpha and beta-diversity of Antarctic meiofauna and start to assess their likely resilience in the context of climate change.

The first aim of this study was to provide a global description of marine Antarctic meiofaunal diversity and community structure in shallow waters, using high throughput sequencing approaches on community DNA. Secondly, to compare Antarctic shallow-water datasets with deep-sea samples taken in the same area (both published and un-published) to identify general diversity trends in freezing habitats and potential depth gradients. A third aspect was to compare the data generated here with those of another metabarcoding study on meiofaunal samples from a mid-temperate region using the same 18 S rRNA region to identify relative levels of biodiversity and whether these were markedly reduced in the Antarctic samples.

## Results

**Figure 1:**
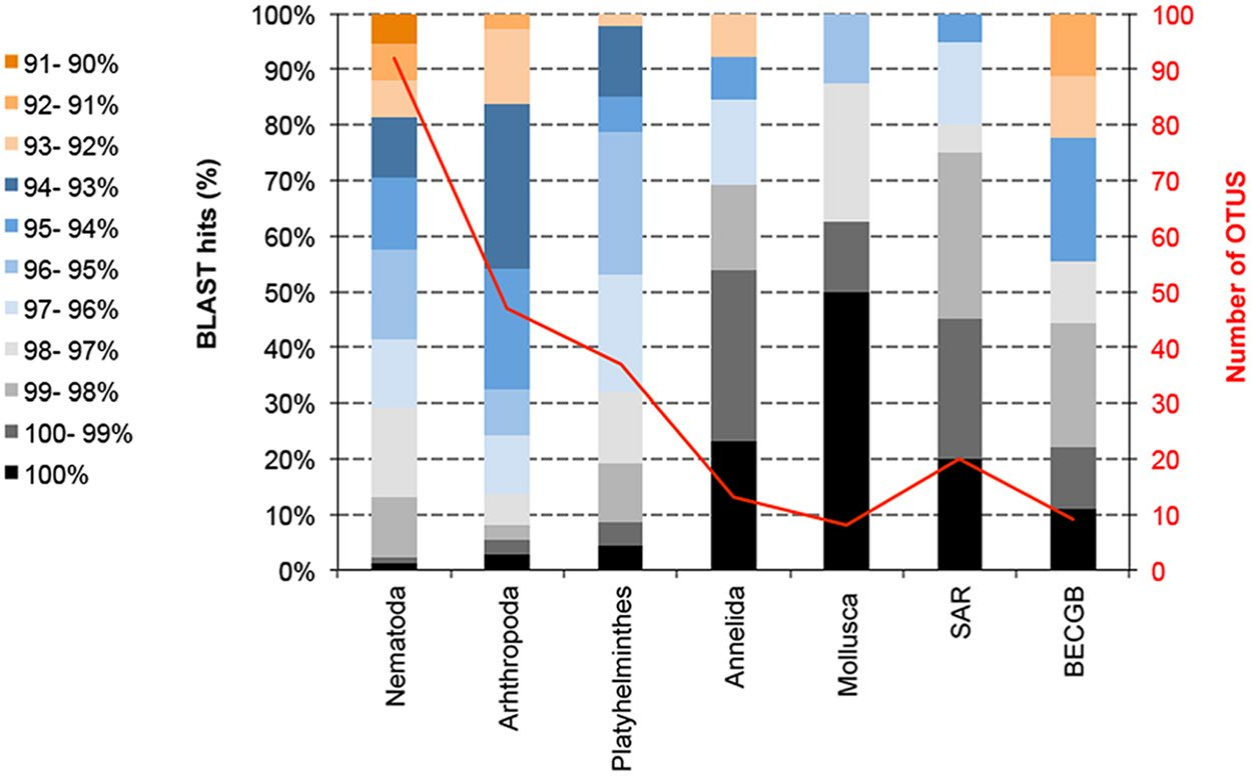
Percent identity to known sequences and number of OTUs found for the main meiofauna phyla retrieved from the Antarctic Peninsula sampled sites. The red full line represents the total number of OTUs found per phyla and the coloured bar represents the percentage identity BLAST match against the SILVA 111 nucleotide database. OTU percentages of BLAST match identity against the SILVA database are shown black (100% BLAST), dark to light grey (100-97% BLAST), light to dark blue (97-93%) and light to dark orange (93-90% BLAST). BECGB: Brachiopoda, Echinodermata, Cnidaria, Gastrotricha, Bryozoa.

**Figure 2:**
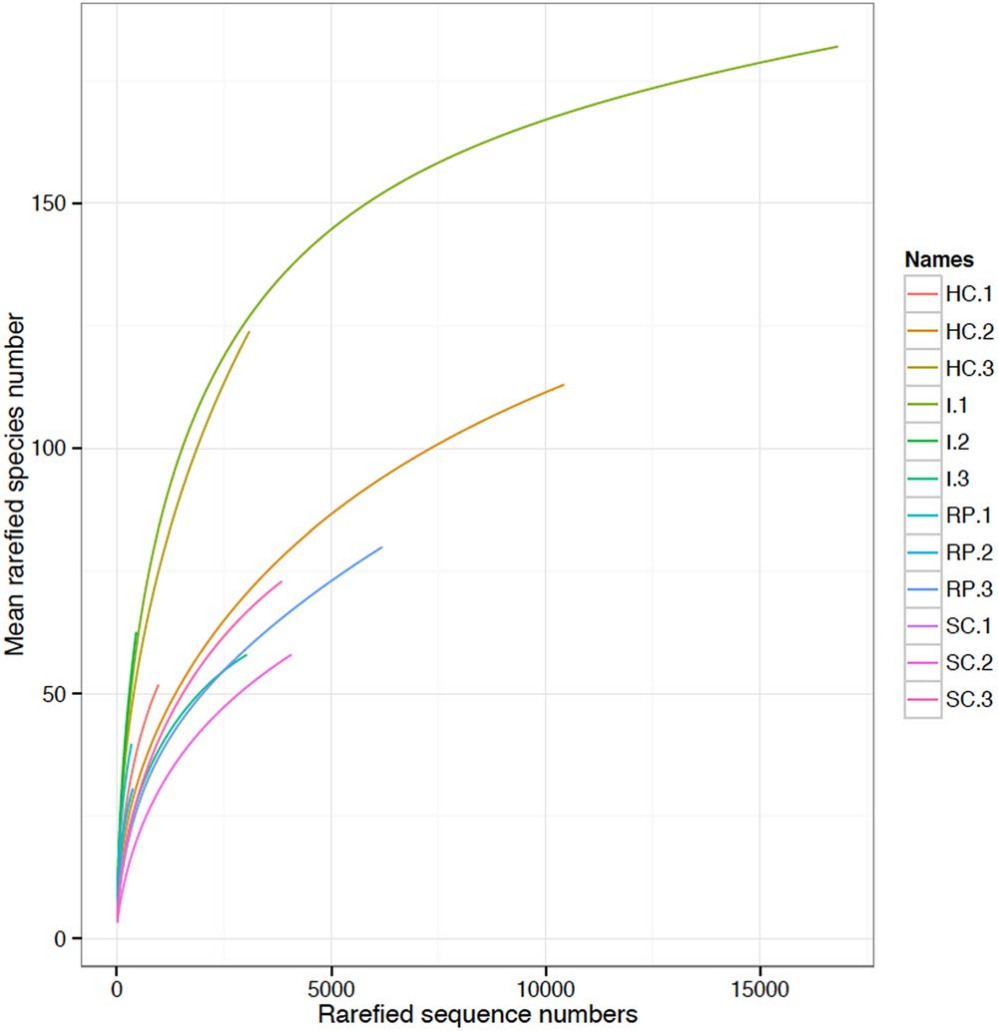
Operational taxonomic unit saturation profiles at 99% sequence similarity level, for the. Antarctic samples collected. Hangar Cove (HC), Islands (I), Rothera Point (RP) and South Cove (SC), where 1–3 represent each sample replicate.

The total number of reads derived from the 454 FLX sequencing platform from the Antarctic Peninsula sampled sites was 61,057; which was reduced to 49,655 reads after filtering and chimera removal. This level of reduction in read numbers was comparable with previous 454 eDNA studies^26, 27^. This particular chemistry introduces higher error rates than the Illumina platform within homopolymer regions due to accumulated light intensity variation, but these reads can be identified and removed *in silico*. Additional reads were removed as they were only present in singletons and through the application of UCHIME, which is known to be a stringent filtering step^27^. Metazoan OTU numbers varied moderately between sample sites with a mean number of 90 OTUs in Hangar Cove (stdv ± 36.09), 48.7 OTUs in Rothera Point (stdv ± 26.05), 87 OTUs in Islands (stdv ± 60.65) and South Cove with mean OTUs number of 47 (stdv ± 24.24). A major proportion of the OTUs from each site (16–31%) were not assigned to any annotated taxa in SILVA database (Table 1). In terms of those taxa with matches in SILVA, the nematodes had the highest OTU numbers among the main phyla, with 92 OTUs followed by the arthropods and platyhelminthes represented by 47 and 37 OTUs respectively **(**Fig. 1). More detailed taxonomy assignments retrieved for each clustered OTU (using a cut-off of 90% to any reference nSSU) showed that the majority (95–98%) of platyhelminth, arthropod and nematod OTUs were not present in the SILVA database (Fig. 1). In total this represented 171 OTUs (30% of OTUs comprising 37671 individual sequences) which may represent un-sampled diversity. The annelids and molluscs, however, had 23% and 50% respectively, of their OTUs with a 100% identity to previously sequenced taxa. The Brachiopoda, Echinodermata, Cnidaria, Gastrotricha and Bryozoa were grouped as BECGB with a total of 9 OTUs where 11% of which had 100% identity matches to previously annotated sequence data. Sampling saturation profiles showed that the sequencing effort was not sufficient to determine the full extent of the diversity for any of the four sampled sites (Fig. 2 **)**. The slope of the OTU rarefaction curves did not approach saturation at 97% cut-off for all the meiobenthic phyla and more specifically for the nematodes, arthropods and even for the platyhelminthes which comprised a low abundance phylum where rarefaction curves tend to converge and reach an asymptote^28^ (Supplementary Figure S1) and therefore the data described here are underestimates.

**Table 1:** Summary data for the sampled areas Hangar (HC), Rothera point (RP), Islands (I) and South Cove (SC) at Rothera in the Antarctic Peninsula. The number of reads before (No reads) and after denoising (QC/CC): QC: quality score; CC: chimera check) and total OTU numbers are shown. OTUs numbers were taxonomically assigned to the eukaryotes and unknown. The latter samples comprised both sequences with no matches in the SILVA reference database and also matches to unannotated environmental samples.

Location	Depth (m)	No Reads	QC/CC	Number of OTUs		
				Eukaryote	Unknown	Total
Hangar	18	18391	14445	116	43	159
Rothera Point	15	8110	6898	85	16	101
Islands	13	23882	20109	127	58	185
South cove	8	5740	8203	76	19	95

**Figure 3:**
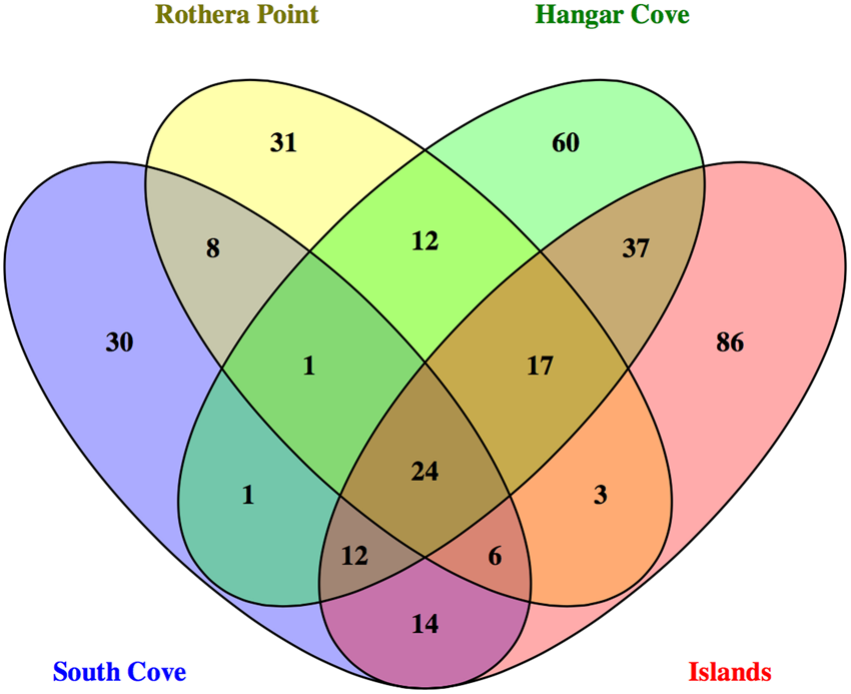
Venn diagram depicting OTUs that are shared or unique to each of the four sampling sites found in the Antarctica meiofaunal shallow waters. Numbers in the diagram represent the number of total OTUs found in the different samples, South Cove (blue), Islands (Red), Rothera Point (yellow) and Hangar Cove (green).

Community composition by number of OTUs did not show significant differences between the sites, with the nematodes totalling ca 30–50 OTUs (Kruskal-Wallis, p = 0.189) followed by the arthropods with ca. 20–30 OTUs (Kruskal-Wallis, p = 0.901), the platyhelminthes with ca. 10–20 (Kruskal-Wallis, p = 0.494), OTUs and the annelids with ca. 3–9 OTUS (Kruskal-Wallis, p = 0.110), found in the Antarctic meiobenthic samples (Supplementary Figure S2). In fact, the majority of the samples showed that 90–100% of the OTUs were shared between sites, with the exception of one of the triplicates of the Islands sample that had approximately 30% of unique OTUs (Fig. 3). Whilst all sites showed globally very similar communities, cluster analysis for taxonomic patterns of meiofaunal communities based on Sørensen similarities of OTU presence/absence data for the combined sites showed two well-defined groups within the Antarctic Peninsula sampling sites (Supplementary Figure S3). The Islands and Hangar Cove were more similar to each other, sharing approximately 20% more OTUs than with South Cove and Rothera Point (data not shown).

**Figure 4:**
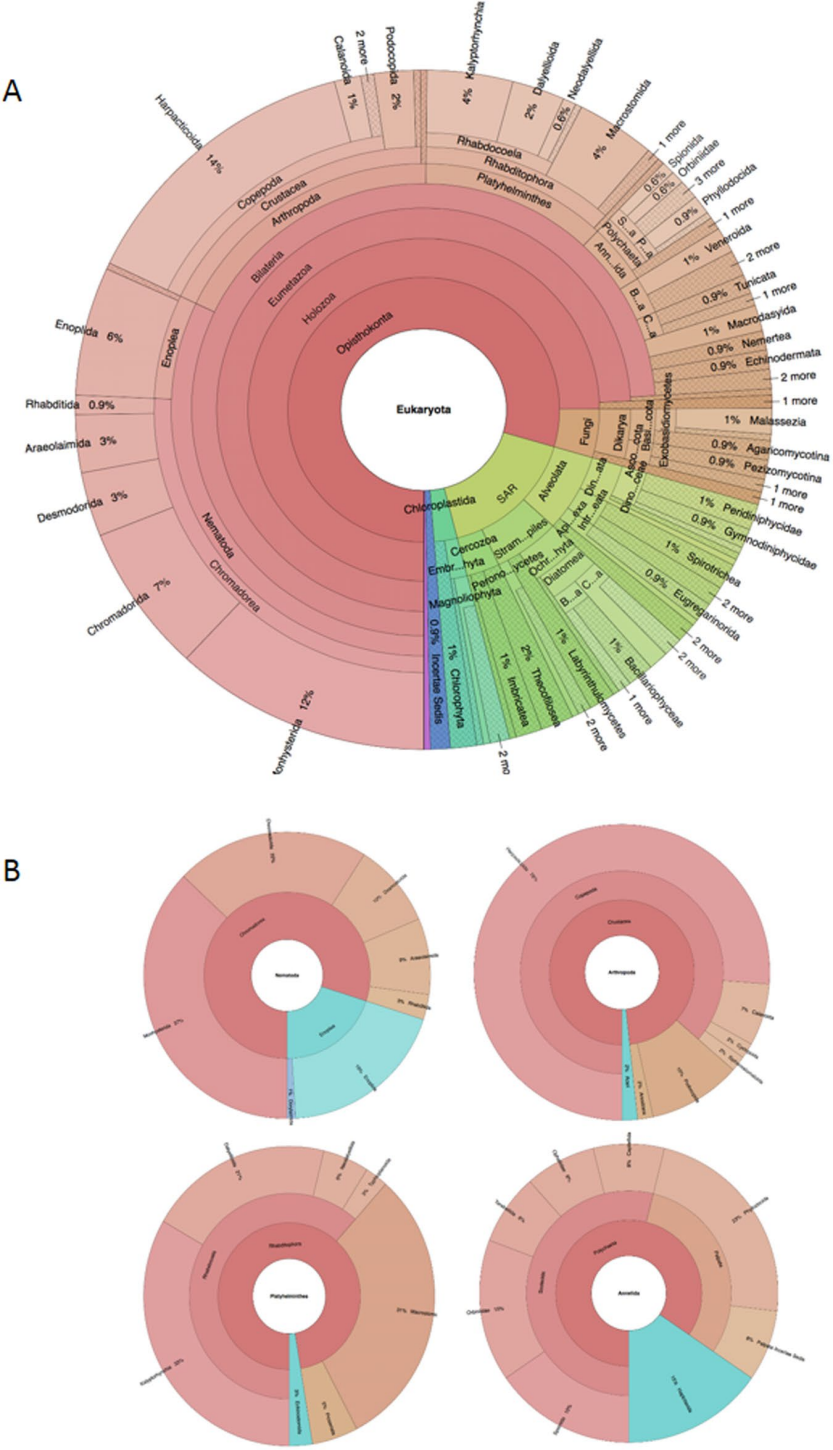
Krona graphical representation of the relative taxonomic contributions (OTU percentages) of the main eukaryotic (**A**) and meiofauna representatives (**B**) found at Rothera Peninsula sampled sites, using taxonomic assignment from SILVAngs 1.5 database at https://www.arb-silva.de/ngs/. Depicted are also OTU percentages of four of the main meiofauna phyla found, the nematodes, arthropods, platyhelminthes and the annelids.

Graphical representation of community composition from all sample sites was visualized with the Krona chart (Fig. 4A). Here, the eukaryotic taxonomic composition of all sites combined showed that the nematodes comprised 32% of the total eukaryotic OTUs. Followed by the arthropods, platyhelminthes and annelids with 18%, 12% and 4% representing the total eukaryotic biodiversity, respectively. Within the nematodes two taxonomic classes predominated: the Chromadorea (80% OTUs) and the Enoplea (20% OTUs) (Fig. 4B, Supplementary Table S1.1–S1.3, Supplementary Material S1). Within these two taxa, Monhysterida (37% OTUs) and Enoplida (19% OTUs) comprised the major proportion of the identifications respectively (Fig. 4B, Supplementary Table S1.1–S1.3). Copepoda dominated the arthropods with 87% of the identified OTUs. The Harpacticoida were particularly abundant at 76% of the Copepoda (Fig. 4b, Supplementary Table S1.1–S1.3, Supplementary Material S1). Outside of the crustaceans, the Acari represented 2% of the arthropod OTUs. The platyhelminthes were mainly represented by with the Rhabditophora (97%) with predominance of the orders Rhabdocoela (62%) and Macrostomida (31%) (Fig. 4b, Supplementary Table S1.1–S1.3, Supplementary Material S1). The annelids were mainly composed of the Polychaeta (85%) and the Haplotaxida (15%). The Polychaeta were dominated by the subclass Palpata (31%) and infraclass Scolecida (54%). The Palpata comprised the Phylodocida order (23%) and taxa with uncertain taxonomic position (Incertae Sedis) (8%). The Scolecida covered five distinct families with the Spionida (15%), Orbibidae (15%), Terebellida (8%), Ophellidae (8%) and the Capitellida (8%) (Fig. 4b, Supplementary Table S1.1–S1.3, Supplementary Material S1), identifications which have been further substantiated by 18 s rRNA molecular barcoding of polychaete samples from shallow-water hard and soft sediment communities near Rothera (Clark, unpublished data).

The shallow-water comparisons with deep-water samples taken from along the Antarctic Peninsula showed very different community compositions (Supplementary Figure S4). Although the annelids and nematodes were found at both depths, they were particularly dominant in the deep-water samples. Shallow-water samples had a much higher percentage of arthropods (or more precisely, copepods). The Nemertea and Hemicordata were essentially only found in the deep samples, with the Cnidaria, Echinodermata and Mollusca more common in the shallows. The difference in community composition was further substantiated by pairwise comparisons of the number of shared OTUs between the different deep-water samples with the combined shallow samples, with the shallow-water sites sharing on average ca. 15% of OTUs with the different deep-water sites (Supplementary Figure S5). It should be noted that comparisons of two of the deep-water sites taken at a similar depth (CTD, 515 m and Laubeuf, 500 m) showed only 20.4% shared OTUs, indicating the patchiness of distributions (similar shallow-water comparisons between the Islands (13 m) and Rothera Point (15 m) showed 26.7% shared OTUs) (data not shown).

## Discussion

This study shows interesting insights into levels of meiofaunal biodiversity in Antarctic sediments, suggesting similar levels of meiobenthic diversity when compared to other marine studies carried out in more temperate regions using the same nSSU gene region^26^, which is higher than expected. Such evidence emerges when comparing the incomplete slopes of the rarefaction curves and OTU numbers obtained here with a previous study on a Scottish temperate benthic ecosystem^26^ using an identical 18 S rRNA gene region, a 97% identity cut-off and the same number of replicates, showing both sites to be very similar (e.g. 540 Antarctic and 650 Scottish meiofauna total OTUs). This evidence is not in line with paradigms of reducing diversity with latitude^29^. It also suggests that Antarctic meiofaunal biodiversity could be as rich and diverse as that found in temperate areas.

This preliminary study reveals that almost all of the main meiobenthic biodiversity is yet to be described, particularly with regard to taxonomic identification and development of associated barcodes, since only 1–4% of our taxa had a full taxonomy match against public databases (Fig. 1). Such low levels of taxonomy assignments are almost certainly the result of the lack of Antarctic species in eukaryotic sequence databases, limited and patchy sampling regimes and the almost total absence of knowledge of Antarctic meiobenthic biodiversity in many taxa^30^. Studies on the benthos around the Antarctic Peninsula have found more than 20% of new families, genera and species, which emphasizes that these habitats contain not only new species records but previously undescribed taxa^3, 31^. For example, more than half of the known gastropods and bivalve mollusc species in the Antarctic have only been found once or twice^30^. Although this level of novelty might seem atypical for such an extensive but harsh environment, it is somehow reasonable that a topographically complex and remote area such as the Antarctic would be bound to contain new species due to the long period of biogeographic isolation via the Antarctic Circumpolar Current, especially if some of these areas have been little or never sampled before^32^.

In this study, the phylogenetic analysis and the taxonomic assignments retrieved from the SILVA database produced four dominant taxonomically distinct metazoan groups, the nematodes, arthropods, platyhelminthes and the annelids (Fig. 4a and b, Supplemental Material S1). These results are supported by previous studies showing that nematodes and Harpaticoid copepods dominate the Antarctic benthos^33, 34^. Additionally, very few studies describe platyhelminthes living within Antarctic sediments possibly because they are commonly known to live in the sea-ice and feed on sea ice diatoms^35^ but may also be explained by a likely high destruction rate of their soft bodies when sampled for physical taxonomic studies. Most annelids found in this study, were dominated by the polychaetes, which tend to be transient meiofauna associated with Antarctic sediments^36^. These data are supported by a macrofaunal (>1 mm) taxonomic study in the same region, which showed a predominance of Arthropods and Annelids (polychaete worms) in the sediments^37^. The more fragile nematode samples were largely identified using molecular techniques, which showed them to be the dominant microtaxa, followed by Arthropods and Platyhelminthes^37^. In our study there were also some identified phyla with very few assigned OTUs (Mollusca, Brachiopoda and Echinodermata). However, given the size fractionation methodology used in this study (<500 μm, >45 μm), these low abundant OTUs would be either traces of larval or very early post-settlement stages or more likely, gut contents of detritivores, cell debris, faeces, pieces of dermis etc. from adult benthic colonisers. Indeed the macrofaunal study showed that molluscs were highly represented, particularly by *Mysella charcoti* and *Aequiyoldia eightsi*, which would have been largely excluded in meiofaunal fractionation^37^. Taxonomy studies in the Southern Ocean^1, 2^ have described a greater number of species than presented in this data set here (for example 524 nematode species compared with our estimate of 140 OTUs). However the fact that we identified such a number of OTUs in shallow waters at four sampling sites, some of which are geographically close (rather than the whole of the Southern Ocean for the 524 species^2^) (Supplementary Figure S2) validates the conclusion that there is still much to discover, especially in the sediments.

While, the four meiobenthic phyla described here are the main representatives found in the benthos anywhere in the world, there will be taxonomic differences in community structures at the species level. This is reflected in trophic features and reproductive strategies, which in the case of the shallow-water meiofauna in Antarctica are adjusted to a cold, highly disturbed and food limiting environment. Stable isotope analyses of meiofaunal communities in Potter Cove, Antarctic Peninsula (latitude -62.235, longitude -58.663) have shown relatively small food webs, based mainly on non-selective deposit feeders, epistrate feeders and a higher proportion of predators^22^. This was substantiated in our study where the taxonomic assignment within the nematodes were dominated by the *Neochromodora*, *Desmolaimus* and *Sabieteria* genera, suggesting that nematode assemblages were mainly composed of deposit feeders and epistrate feeders, which can minimize interspecific competition. There was also a proportion of Enoplea nematodes that are known to be predators/omnivores. Such different feeding strategies will alleviate species competition to available food^38, 39^. Molecular analyses, such as metabarcoding used here, allow the identification of previously unknown levels of biodiversity^20^ and enable studies that would otherwise not be possible in such detail using other methodologies. In this study, for each of the main meiobenthic phyla (nematodes, arthropods, platyhelminthes and annelids) (Supplemental Material S1) there were some well-supported clades, particularly in the nematodes and nematodes, where OTUs assigned to the same genus, could potentially comprise species complexes. However without further molecular and taxonomic analysis, these would be difficult to define, but would be highly likely^40^.

Clustering of sites according to community composition similarity revealed two well-defined groups (Supplementary Figure S3). The first composed of South Cove (8 m depth) and Rothera Point (15 m depth), represented virtually adjacent sites and thus their clustering confirmed the similarity of their meiobenthic community assemblages. The second cluster was comprised of Hangar Cove (18 m depth) and the Islands (13 m depth). This is substantiated by the macrofaunal study which showed significant patchiness and differences between different coves^37^. South Cove and Rothera Point are more exposed areas with smaller levels of sediment than Hangar Cove or the Islands and likely subject to different current patterns within Ryder Bay and also increased iceberg scour. Generally, replicates of each ecological location always clustered together and thus the combined replicate meiobenthic samples accurately reflected alpha diversity from the Antarctic Peninsula, as shown previously in similar studies in more temperate areas^41, 42^. Meiobenthic community composition can be extremely variable even within small spatial scales^21, 26, 43–46^. Local patchiness and structure within these communities is probably a consequence of a combination of several biotic and abiotic factors^41, 42^. Similar to global observations, sediment type and grain size play large roles in structuring Antarctic communities^21, 23, 37^, with the additional factors of food supply, which influences species richness and ice disturbance^23^. Glacial retreat, ice shelf collapse and the increasing frequency of iceberg scour are significantly impacting the Antarctic benthos, particularly the more shallow waters^12, 21–23, 47^. Species return is largely dictated by motility, with the three main methods of return being locomotion, advection by storms and larval re-colonisation^48^. Overall, only the most resilient animals (probably r-selection species) are able to regularly resist such local impacts and prosper in these harsh environments^49^. Studies on Antarctic sediments have shown that nematodes are able to resist and survive in such harsh conditions, namely after ice disturbance nematode communities are very little impacted^33^, which again reflects their dominance within the benthos described here.

The shallow-water data were also compared to six deep sea samples from the Peninsula region (Supplementary Figures S4 and S5). There was a clear difference in phyla composition with the deep sea sites dominated by nematodes and the shallow by both nematodes and arthropods (or more specifically copepods). These data confirm existing published information on the differences between shallow and deep meiofauna and fit with previous analyses showing biodiversity patterns associated with sediment type and grain size. The shallow samples comprised coarser grains, which are a more favourable habitat for copepods, whilst the deeper sites comprised more fine sediments (mud) suitable for nematodes, as noted in previous studies^20, 23, 37^. What was interesting to note was the relatively small overlap in shared OTUs between the shallow and deep samples (Supplementary Figure S5). Because of the way the OTUs were clustered at 97% similarity, “same OTU” in these comparisons may represent the same genus or family, but is unlikely to be the same species in all OTUs^50, 51^. However, the 97% cut-off for OTU clustering is a known proxy for most meiofaunal studies. Although the physical processing of the shallow and deep samples was slightly different, the rest of the process was identical (primers used in the initial amplification reactions and processing of the data such as removal of non-metazoan OTUs from the comparisons between the two studies) and contributed to standardising the data comparison. Moreover, the higher sensitivity for extracellular DNA of the methods used in the deeper sediments should have actually increased the amount of overlap between shallow and deep due to sedimentation, yet very limited overlap was observed.

**Figure 5:**
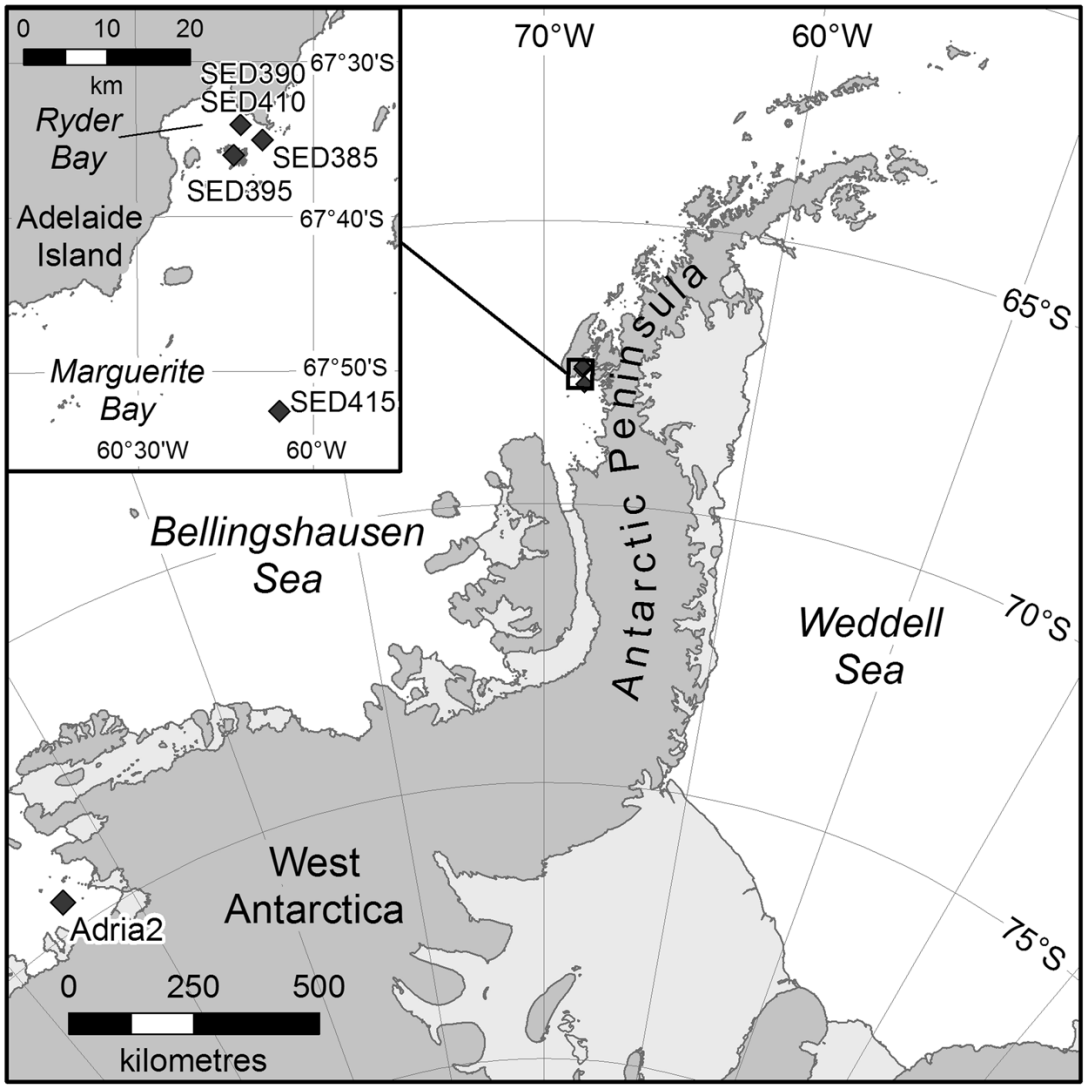
Map showing the main sampling sites along the Antarctic Peninsula, with finer detail of the deep-water sites in Ryder Bay. SED 385 is closest to Rothera Research Station and the sites of the four shallow-water sediment-sampling sites (not shown at this scale). Maps made in-house at BAS using ArcGIS v10.1 by the Mapping and Geographical Information Centre (MAGIC).

This lack of overlap between shallow and deep sites is particularly interesting as the deep CTD samples were quite close to all the shallow sites (Fig. 5) and the CTD sampling site was at the bottom of the Marguerite Bay trough. One could expect all the OTUs from the shallow sites to passively sink/disperse to the deepest point and this clearly does not happen or the conditions at depth select against shallow dwelling species. This depth zonation has been shown previously^20, 34^ and as yet, there is not a clear answer as to whether there is true depth zonation of meiofauna or whether the shallow DNAs are simply too diluted or degraded by the time they reach the deep. Further to this, more sampling effort would be needed to clarify meiofauna zonation patterns since the rarefaction curves for the sampled Antarctic areas remained incomplete and thus community composition and diversity levels are yet to be determined. The question of faunal exchange between deep and shallow waters is the subject of much debate and may vary according to species ecology, but is a clear area for further research^23^. Interestingly even after five years, the meiofaunal communities of the innermost embayments of Larsen B (at 242–427 m depth) were still much more similar to those from the deep sea (800–4000 m), than shallow shelf communities suggesting that perhaps such zonation does exist. In addition these data show that recolonisation and restructuring of meiofaunal communities is not rapid and less likely to be subject to the rapid shifts as seen in motile megabenthic communities^21–23, 52^. Because they are less motile, they may be forced to adapt and thus the signals of change may be clearer in these smaller species^6^. However, what is clear in both shallow and deep-sea Antarctic samples is the high levels of undiscovered taxa and potentially high levels of biodiversity, in what are often described as species-poor regions of the globe.

## Conclusions

Our results suggest that meiofaunal biodiversity in the shallow waters of the Antarctic is at least similar to that of temperate regions. The Antarctic comprises ca. 10–11% of the World’s continental-shelf-area and the total number of validated marine species (mega- and macrofauna) described for the Southern Ocean exceeds 8,000 species, with at least as many more expected^1, 2^. Antarctic meiofaunal descriptions are relatively few to date and have concentrated on taxonomic characterisation. Taxonomically classification of all species is often not practical due to the lack of suitably qualified taxonomists and the sheer volume of work required, thus environmental high throughput sequencing enables faster surveys into understanding biodiversity, albeit providing a slightly different type of data. It also facilitates studies that would otherwise be impossible particularly when applied to bulk environmental samples containing small and easily damaged taxa obtained from inhospitable regions^27^. The study described here showed that much of the Rothera meiofaunal biodiversity is yet to be described, as no plateau was reached from the rarefaction curves and most OTUs could not be annotated with confidence using the public databases. It also shows that the genomic variability of the 18 S rRNA gene can effectively be used to reflect the high but also intangible level of biodiversity even in such a relatively small dataset used in this study and that the methodology is highly tractable for more detailed samplings in the future. These will enable us to gain a more accurate understanding of patchiness and adaptation of meiobethic communities to different environments. This approach may be particularly useful for detecting molecular taxonomic signatures of response to climate change not only in terms of gradual sea warming and acidification, but also the emergence of new habitats resulting from anthropogenic change.

## Material and Methods

### Sample collection

Sediment samples were collected in triplicate at different depths in four different sites near Rothera Station, Adelaide Island on the Antarctic Peninsula (Fig. 5). Sampled areas comprised the Islands (67°35.6′ S, 68°15.1′ W, 13 m depth), Hangar Cove (67°33.8′ S, 68°07.6′ W, 18 m depth), South Cove (67°34.2′ S, 68°7.9′ W, 8 m depth) and Rothera Point (67°34′19′S, 68°6′44′W, 15 m depth). Samples were collected using a standard corer methodology. All samples were immediately fixed in 500 ml storage pots containing 300 ml of DESS (20% DMSO and 0.25 M disodium EDTA, saturated with NaCl, pH 8.0)^53^. The meiofaunal size fraction was mechanically separated from the sand and concentrated by decanting five times with filtered tap water through a 45 µm filter. Subsequent separation from fine silt was achieved by repetitive centrifugation in 1.16 specific gravity (sg) LUDOX-TM solution^54^. Following centrifugation, each sample was retained on a distinct mesh sieve which was then folded, sliced and placed in a 15 ml falcon tube and kept at −80 °C until DNA extraction. Samples were lysed overnight at 55 °C in lysis buffer (100 mM Tris-HCl, pH 7.5; 100 mM NaCl; 100 mM EDTA; 1% SDS, 500 µg/ml proteinase K), assisted by spinning wheel mixing, and DNA extracted with the QIAamp DNA Blood Maxi Kit (Qiagen) following the manufacturer’s protocol^26^.

### Primer design and PCR

Due to the extreme sensitivity of this methodology, all PCR and DNA extractions were carried out in separate rooms and recommended eDNA practices were applied to avoid cross-contamination between samples. The primers were SSU_ F04 primer (GCTTGTCTCAAAGATTAAGCC) and SSU_R22mod (5′-CCTGCTGCCTTCCTTRGA-3′) were used to amplify approximately 450 bp of the V1–V2 regions of the nuclear small subunit rDNA (18 S rDNA)^20^. Fusion primers, PCR amplification and 454 Roche sequencing were performed as described previously^26, 27^. Specifically, PCR amplification of the specified nSSU region was performed using 1 µl of genomic DNA template (1:500 dilutions) in 3 × 40 µl independent reactions with Pfu DNA polymerase (Promega). PCR conditions involved a 5 min denaturation at 95 °C, then 35 cycles with 1 min at 95 °C, 45 s 57 °C, 3 min 72 °C and a final extension of 10 min at 72 °C. Negative controls (ultrapure water only) were included for all amplification reactions. Subsequently, triplicates of PCR products were visualized and the expected 450 bp fragment was purified (QIAquick Gel Extraction Kit, Qiagen) in an agarose gel and quantified using the Agilent Bioanalyser 2100. All purified PCR products were diluted to the same concentration, pooled together to create one metagenetic sample/ library and sequenced in one direction (A-Amplicon) on half a plate of a Roche 454 GSFLX platform (2 × 250 bp) at the Centre for Genomic Research, Liverpool. For full details of replicated PCRs and associated MID tags, see Supplementary Table S2.

### Data analysis and generation of OTUs

Raw sequence reads were filtered and denoised using FlowClus^55^. The filtering criteria included truncating reads prior to the first ambiguous base, the reverse primer, or a window of 50 bp whose average quality score was less than 25.0. Any reads shorter than 200 bp or longer than 600 bp were eliminated. For the denoising step, in which pyrosequencing errors were corrected by clustering the flowgrams, a constant value of 0.50 was used for the denoising distance^56^. After denoising, PCR chimeras were removed using UCHIME^57^ (Supplementary Table S2). The remaining reads were then analysed using QIIME^58^. They were clustered into OTUs at 97% sequence similarity using UCLUST^59^ (pick_otus.py), and taxonomic assignment was performed using the Silva 111 database^60^ (assign_taxonomy.py), which uses uclust. The UNCLUST consensus taxonomy assigner retrieves the maximum assigned matches for each query sequence. It then assigns the most specific taxonomic label that is associated with at least min_consensus_fraction of the matches. It is acknowledged that the threshold used for the OTU clustering at 97% similarity might cluster genus or family from the same taxa, as intra-specific variability will differ across many taxa/species. However, this cut-off is known as proxy for most meiofauna species^50^, but cut-offs such as 99% have also been justified as a proxy for some nematode species in more targeted studies^51^. For direct ecological comparisons among samples with different read numbers, the percentage of reads in each sample was used instead of read counts and downstream analyses targeted main representatives within meiofauna phyla occupying the Antarctic Peninsula sediment habitats^42^.

### Data Deposition

All sequence reads have been deposited in the European Nucleotide Archive (ENA) with accession number ENA: PRJEB1952.

### Diversity and community analysis

Rarefaction curves were generated with EstimateS 8.2.0 software^61^ using the Chao1 richness estimator; nonetheless other richness estimators were tested (ACE, Chao1, Jackknife1 and Bootstrap) and yielded similar results. Sørensen’s similarity coefficient among samples was computed based on a presence/absence similarity matrix and was used to create cluster dendrograms with 50 random starts, using primer 6^62^. Using the same software, a similarity profile (‘SIMPROF’) permutation test, was performed on group-average cluster analysis to test whether the meiobenthic samples differ from each other. In order to further test for significant differences in community composition among sampling sites, a permutational multivariate analysis of variance (‘PERMANOVA’) was performed. Analyses were based on Sørensen’s similarity coefficient on untransformed data of an OTU presence/absence matrix over the four sampled sites, with 1000 permutations. Further comparisons between the Antarctic and a Scottish study^26^ were performed to illustrate possible differences between the numbers of meiofauna OTUs found per phyla in the two habitats. In order for the two studies to be as comparable as possible, all analysis were performed using triplicated samples, similar 18 S gene regions and using the same OTU clustering threshold of 97%. Antarctic eukaryotic OTUs retrieved from the data analysis were used in a Neighbour-Joining (NJ) phylogeny reconstruction to confirm the taxonomic assignments (Supplemental material S6). Taxonomic contributions using total OTU proportions were visualized using Krona graphs, plotted using the Krona web interface software^63^ and a non-parametrical statistical test was performed (Kruskal-Walis) to check if number of OTUs per replicated sample site were significantly different^.^ Taxonomic assignment for this purpose was also performed using SILVAngs 1.5 database at https://www.arb-silva.de/ngs/.

### Comparison with deep sea samples

Comparisons of OTUs were made with deep sea meiofaunal data from samples taken along the Antarctic Peninsula^20^ and comprise SED 415 (Laubeuf Fjord) (500 m) (67°52.583 S 68°5.842′W), SED 390 and SED 410 (duplicate CTD samples at the same site and depth) (515 m) (67°35′6.57 S 68°12′17.38 W), SED385 (390 m) (67°35′6.16″S 68°8′35.42″W), SED395 (off Anchorage Island) (290 m) (67°36′5.23″S 68°13′29.75″W) with an additional sample denoted Adria2 (1120 m) (74°29′.00 S 104°25′.00 W) kindly provided by Holly Bik and Adrian Glover (data unpublished) (Fig. 5). Published data were obtained from direct extractions of minimal amounts of frozen sediments^20^ while the data from the additional sample “Adria2” was processed using the same methodology (DESS fixed samples, meiofauna isolated from sediments and then DNA extraction) as the shallow-water data presented here.

## Electronic supplementary material

Supplementary Information
