# Supplementary material for: Revealing higher than expected meiofaunal diversity in Antarctic sediments: a metabarcoding approach
Source: Sci Rep. 2017 Jul 21;7:6094. doi: 10.1038/s41598-017-06687-x (PMC5522477; doi:10.1038/s41598-017-06687-x)
Supplement: Supplementary file 1 — Supplementary Information [file 41598_2017_6687_MOESM1_ESM.pdf]

Revealing higher than expected meiofaunal diversity in Antarctic sediments: a metabarcoding approach

Supplementary Information

Fonseca VG[1], Sinniger F[2], Gaspar JM[3], Quince C[4], Creer S[5], Deborah M Power[6] , Lloyd S Peck[7], Melody S Clark [7]

[1]Zoological Research Museum Alexander Koenig (ZFMK), Centre for Molecular Biodiversity Research, Bonn, Germany.

[2]Sesoko Station, Tropical Biosphere Research Center, University of the Ryukyus, 3422 Sesoko, Motobu, Okinawa, 905-0227 Japan.

[3]Computational Biology Institute, George Washington University, Ashburn, Virginia, USA.

[4]Department of Microbiology and Infection, Warwick Medical School, University of Warwick, Coventry, CV4 7AL, UK.

[5]Molecular Ecology and Fisheries Genetics Laboratory, School of Biological Sciences, Bangor University, Gwynedd, LL57 2UW, UK.

[6]Centro de Ciencias do Mar, Universidade do Algarve, Campus de Gambelas, Faro, 8005-139, Portugal

[7]British Antarctic Survey, Natural Environment Research Council, High Cross, Madingley Road, Cambridge, CB3 0ET, UK

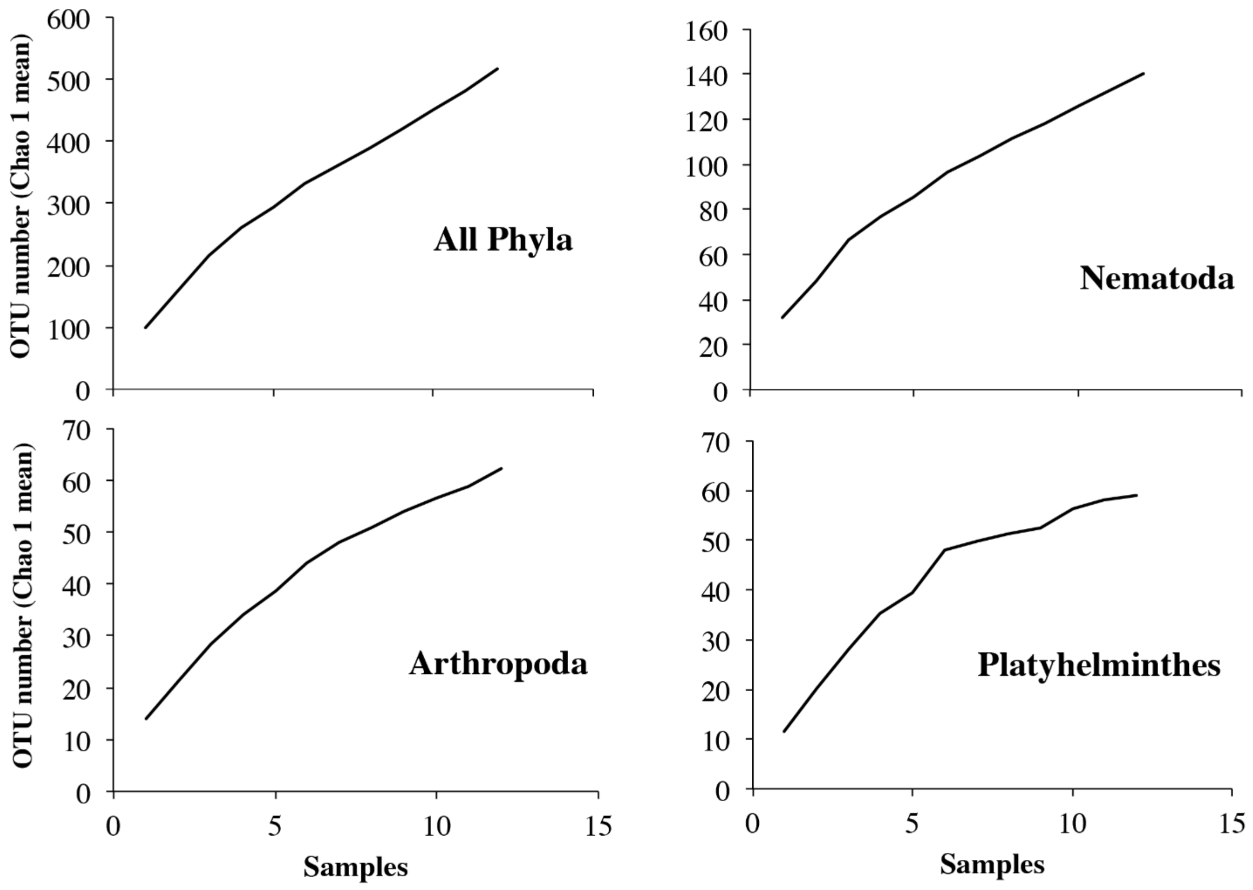

Supplementary Figure S1: Rarefaction curves of the Chao 1 diversity estimator. Plots are shown for all phyla, Nematoda, Arthropoda and Platyhelminthes at 97% identity OTU cut-off for all the Antarctic Peninsula sampled sites samples. Curves were estimated from 100 randomizations, without replacement, using EstimateS, version 8.2.0.

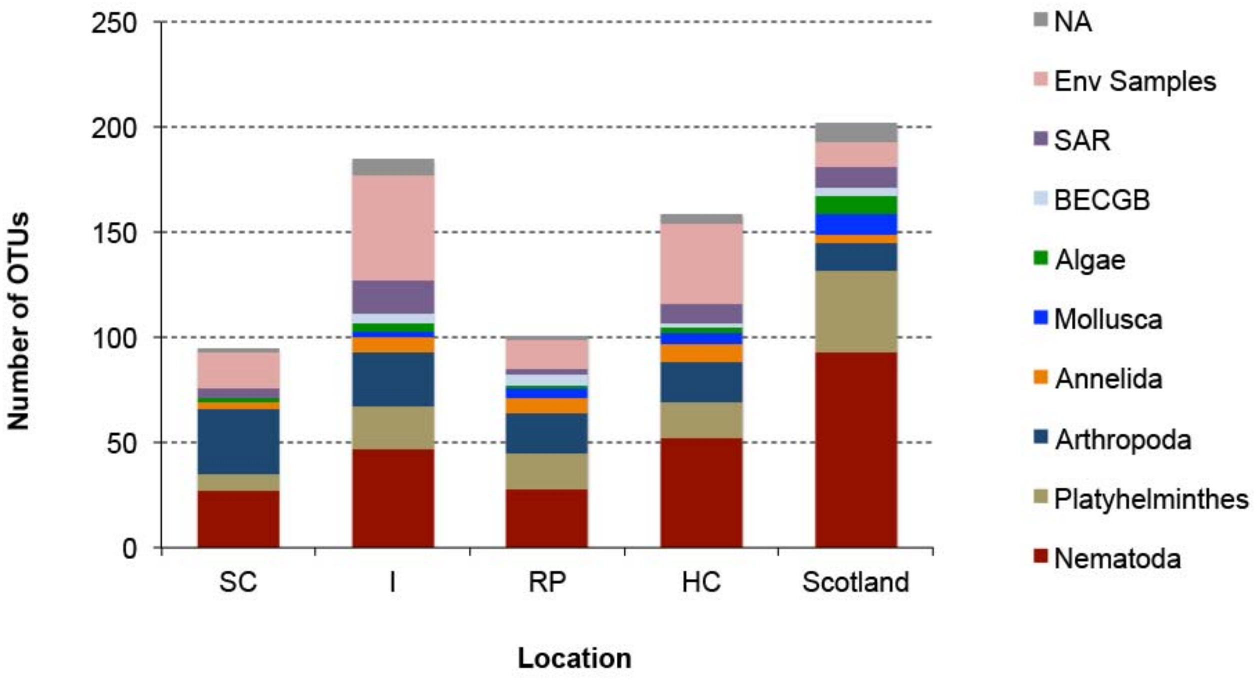

Supplementary Figure S2: Community composition for the Antarctic sampled areas Hangar Cove (HC), Rothera Point (RP), Islands (I), South Cove (SC) and also for the Scottish sampled site[26]. Taxonomy assignment was performed using the SILVA database and the number of total OTUs for each sample site is shown (triplicates were merged per sample site).

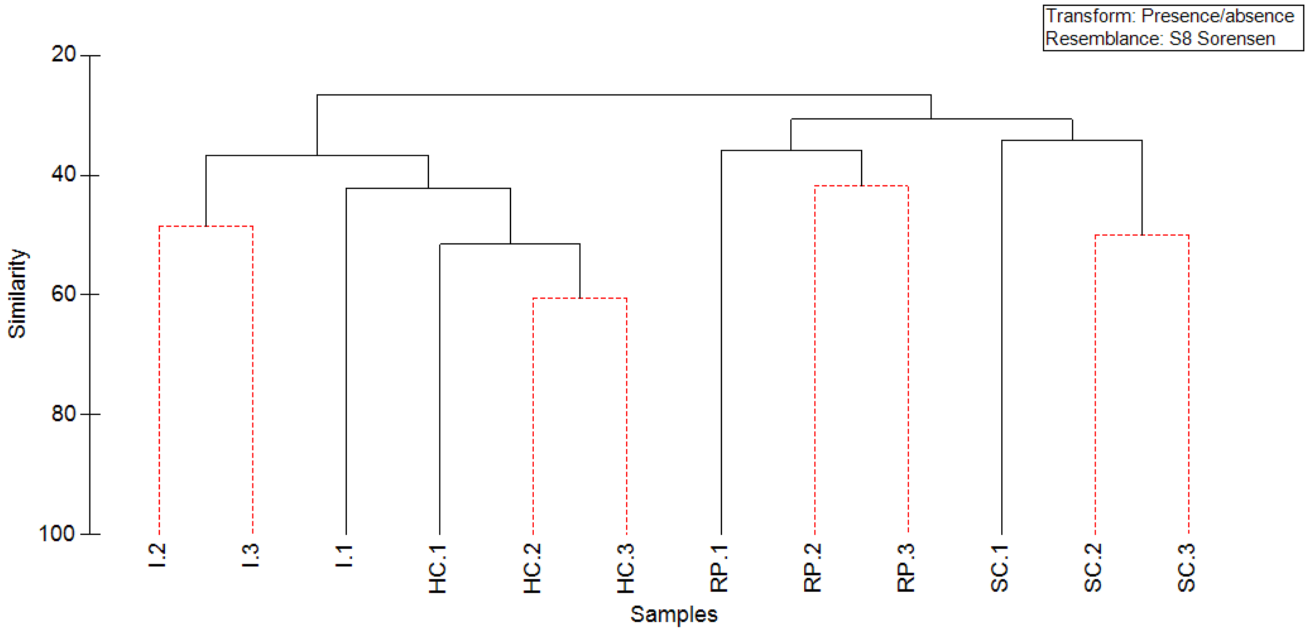

Supplementary_Figure_S3: Cluster analysis for taxonomic patterns of meiofaunal communities based on Sørensen similarities of OTU presence/absence data for the combined sites. In the dendrogram, black solid lines represent samples sharing a significant similarity profile with a SIMPROF analysis.

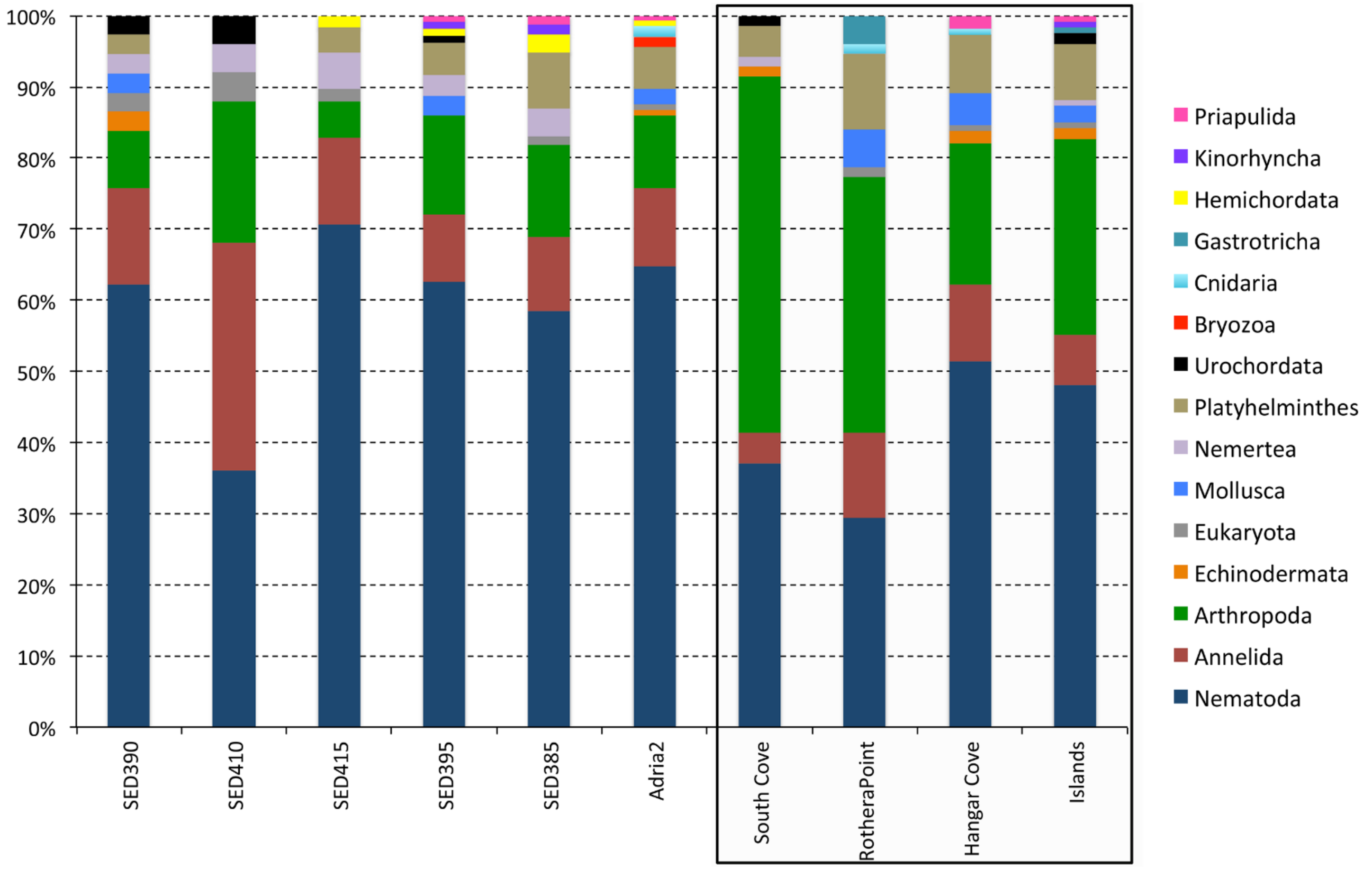

Supplementary Figure S4: Community composition for the shallow and deep-water samples. The shallow-water samples are highlighted within a border. Taxonomy assignment was performed using the SILVA database with the percentage of OTUs per phyla shown in all sample sites.

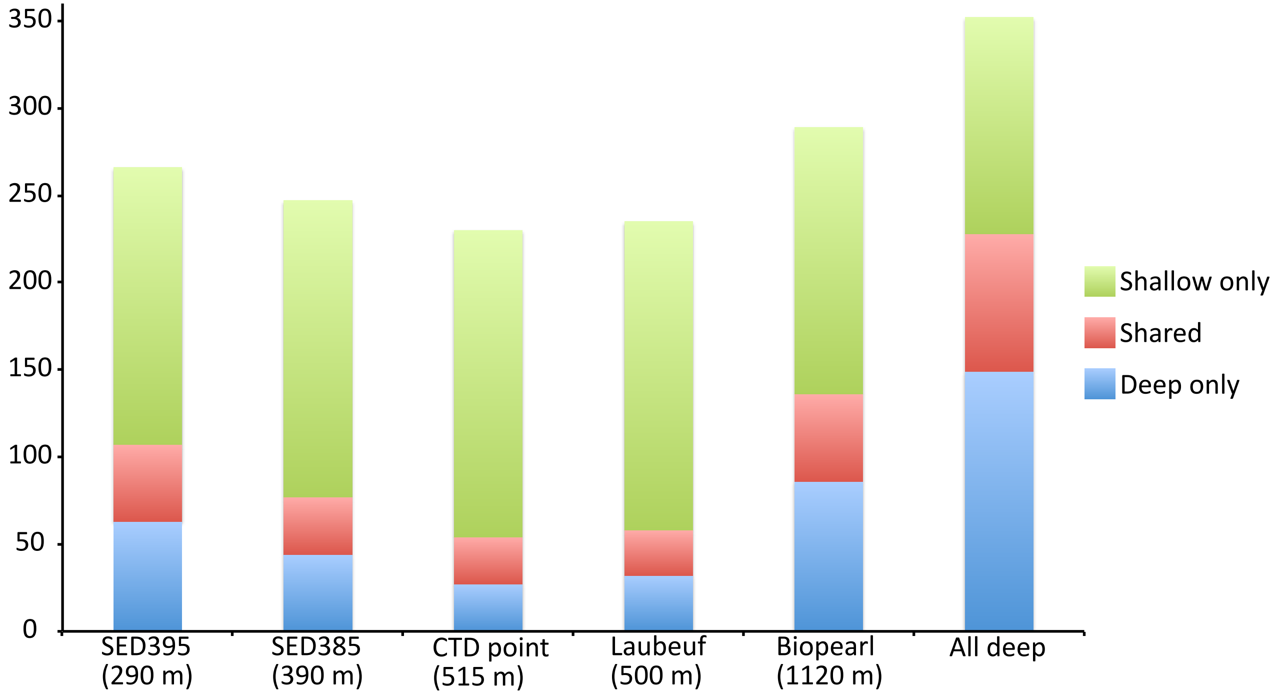

Supplementary Figure S5: Overlap of metazoan OTUs between merged shallow samples and deep samples (individual and merged). Values are based on presence/absence data, with a total of 203 distinct metazoan OTUs found in all shallow samples and between 54 and 136 distinct OTUs in each of the deep samples for a total of 228 different deep metazoan OTUs.

Supplementary Material S1:

Fonseca et al. "**Revealing higher than expected meiofaunal diversity in Antarctic sediments: a metabarcoding approach**"

Supplementary analysis

Method

All Eukaryotic OTUs retrieved from the data analysis were used to confirm the taxonomic position and community composition within the main eukaryotic metazoan found, using a Neighbour-Joining (NJ) phylogeny reconstruction, 500 bootstrap replications and the Kimura 2-parameter pairwise distance model. The analysis was performed using the software Mega7 (Kumar *et al.*, 2016) and illustrated via a phylogenetic tree produced using the Interactive Tree of Life iTOL tool (Letunic & Bork, 2007).

Result

Phylogenetic analysis of the total Eukaryotic OTUs further confirmed the presence of five taxonomically distinct phyla groups, the Nematoda, Arthropoda, Platyhelminthes, Annelida and the SAR supergroup (Starmenopiles, Alveolata and Rhizaria) and all phylogenetic clusters were supported by strong to moderate bootstrap values (Figure S1). OTUs assigned to Fungi were removed from the analysis and the Chloroplastida OTUS (ALGAE) were used as an out-group (Figure S1). The Arthropoda cluster had a strong bootstrap support but it also showed a smaller independent cluster comprised mainly of the Ostracoda class (Figure S1). Here, three Echinodermata OTUs, two Kynorincha OTUs and one Mollusca OTU also sub-clustered. The Mollusca (7 OTUs) and Gastrotricha (4 OTUs) clustered inside the Annelida phyla. Within the SAR supergroup the Rhizaria (Cercozoa) also showed an independent phylogenetic sub-cluster, whereas the Stramenopiles and Alveolata clustered concurrently (Figure S1).

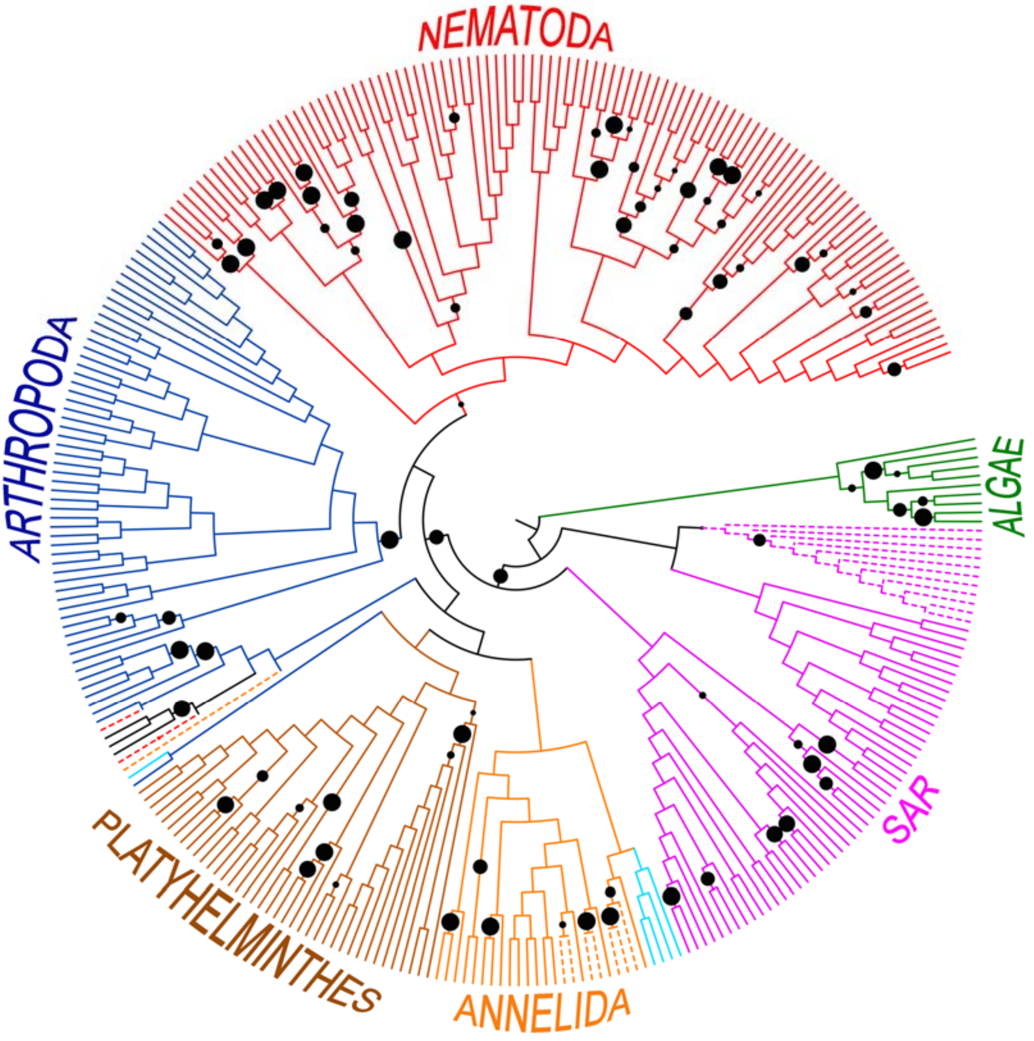

Figure S1- Phylogenetic tree of all Eukaryotic OTUs using a Neighbour-Joining analysis based on the Kimura 2-parameter model. Black symbols at nodes represent the corresponding range of bootstrap support values, from the smallest (75% support) to the largest (100% support). Five main distinct phylogenetic groups were formed the Nematoda, Arthropoda, Platyhelminthes, Annelida and the SAR supergroup (Starmenopiles, Alveolata and Rhizaria). The Rhizaria from the SAR supergroup is depicted in dash-purple. Other phyla are also clustered, the Mollusca (dash-orange), Kynorincha (dash-red), Gastrotricha (light blue) and Echinodermata (solid black). The outgroup is the ALGAE green cluster. SILVA database was used for OTU taxonomy classification.

Supplementary Table S1.1- Closest BLAST matches of Operational Taxonomic Units (OTUs) retrieved from Rothera sample sites, assigned to Nematoda, up to genus or species levels (Description) using SILVA 1.11 database. Depicted are the public accession numbers (AcNumber), BLAST identity percentage against SILVA (BLAST % ID), Phylum and other Taxa ranking.

OTU#	AcNumber	BLAST % ID	Phylum	Taxa Rank	Description
denovo208	gb|AY593940.1|	94,5	phylum:Nematoda	class:Chromadorea	Achromadora cf terricola
denovo219	emb|AJ966473.1|	91,17	phylum:Nematoda	class:Chromadorea	Anaplectus sp.
denovo150	gb|HM564638.1|	98,53	phylum:Nematoda	class:Enoplea	Anticoma sp.
denovo169	gb|HM564638.1|	96,19	phylum:Nematoda	class:Enoplea	Anticoma sp.
denovo46	gb|JN968252.1|	100	phylum:Nematoda	class:Enoplea	Aporcelaimellus sp.
denovo165	gb|KF935309.1|	96,99	phylum:Nemertea	class:Enopla	Argonemertes australiensis
denovo166	gb|FJ040461.1|	96,03	phylum:Nematoda	class:Chromadorea	Axonolaimus sp.
denovo310	gb|FJ040461.1|	92,36	phylum:Nematoda	class:Chromadorea	Axonolaimus sp.
denovo88	emb|AJ966476.1|	92,68	phylum:Nematoda	class:Enoplea	Bathylaimus assimilis
denovo159	gb|AY854218.1|	94,44	phylum:Nematoda	class:Chromadorea	Calomicrolaimus parahonestus
denovo160	gb|AY854218.1|	94,97	phylum:Nematoda	class:Chromadorea	Calomicrolaimus parahonestus
denovo294	gb|AY854218.1|	98,14	phylum:Nematoda	class:Chromadorea	Calomicrolaimus parahonestus
denovo281	gb|JN968284.1|	90	phylum:Nematoda	class:Chromadorea	Calomicrolaimus sp.
denovo336	gb|JX678599.1|	95,62	phylum:Nematoda	class:Chromadorea	Camacolaimus sp.
denovo93	gb|EF591327.1|	97,94	phylum:Nematoda	class:Chromadorea	Camacolaimus sp.
denovo170	gb|HM564544.1|	98,83	phylum:Nematoda	class:Enoplea	Chaetonema sp.
denovo38	gb|JN968217.1|	91,88	phylum:Nematoda	class:Chromadorea	Daptonema sp.
denovo298	gb|JN968217.1|	91,91	phylum:Nematoda	class:Chromadorea	Daptonema sp.
denovo304	gb|EF591333.1|	95,53	phylum:Nematoda	class:Chromadorea	Desmolaimus sp.
denovo275	gb|EF591333.1|	97,63	phylum:Nematoda	class:Chromadorea	Desmolaimus sp.
denovo138	gb|EF591333.1|	95,79	phylum:Nematoda	class:Chromadorea	Desmolaimus sp.
denovo65	gb|EF591333.1|	96,59	phylum:Nematoda	class:Chromadorea	Desmolaimus sp.
denovo184	gb|EF591333.1|	97,63	phylum:Nematoda	class:Chromadorea	Desmolaimus sp.
denovo267	gb|EF591333.1|	94,23	phylum:Nematoda	class:Chromadorea	Desmolaimus sp.
denovo75	gb|EF591333.1|	97,63	phylum:Nematoda	class:Chromadorea	Desmolaimus sp.
denovo8	gb|EF591333.1|	94,74	phylum:Nematoda	class:Chromadorea	Desmolaimus sp.
denovo195	gb|EF591333.1|	94,47	phylum:Nematoda	class:Chromadorea	Desmolaimus sp.
denovo178	gb|EF591333.1|	95,01	phylum:Nematoda	class:Chromadorea	Desmolaimus sp.
denovo321	gb|EF591333.1|	93,79	phylum:Nematoda	class:Chromadorea	Desmolaimus sp.
denovo76	gb|FJ182217.1|	97,63	phylum:Nematoda	class:Chromadorea	Draconema japonicum
denovo168	gb|AY854193.1|	98,66	phylum:Nematoda	class:Enoplea	Enoploides brunettii
denovo42	gb|HM564545.1|	98,49	phylum:Nematoda	class:Enoplea	Halalaimus sp.
denovo330	gb|HM564479.1|	98,5	phylum:Nematoda	class:Enoplea	Halalaimus sp.
denovo84	gb|FJ040458.1|	93,18	phylum:Nematoda	class:Chromadorea	Leptolaimus sp.
denovo209	gb|FJ040458.1|	93,18	phylum:Nematoda	class:Chromadorea	Leptolaimus sp.
denovo97	gb|FJ040458.1|	93,07	phylum:Nematoda	class:Chromadorea	Leptolaimus sp.
denovo81	gb|FJ040458.1|	90,84	phylum:Nematoda	class:Chromadorea	Leptolaimus sp.
denovo124	gb|FJ040458.1|	94	phylum:Nematoda	class:Chromadorea	Leptolaimus sp.
denovo149	gb|JF293035.1|	98,45	phylum:Nemertea	class:Anopla	Lineus torquatus
denovo231	gb|EF591337.1|	92,86	phylum:Nematoda	class:Chromadorea	Linhomoeidae sp.
denovo314	gb|JN968218.1|	93,88	phylum:Nematoda	class:Chromadorea	Metadesmolaimus sp.
denovo110	gb|AY854210.1|	97,89	phylum:Nematoda	class:Chromadorea	Neochromadora
denovo299	gb|AY854210.1|	97,36	phylum:Nematoda	class:Chromadorea	Neochromadora
denovo328	gb|AY854210.1|	96,31	phylum:Nematoda	class:Chromadorea	Neochromadora
denovo198	gb|AY854210.1|	96,57	phylum:Nematoda	class:Chromadorea	Neochromadora
denovo252	gb|AY854210.1|	98,29	phylum:Nematoda	class:Chromadorea	Neochromadora
denovo133	gb|AY854210.1|	95,51	phylum:Nematoda	class:Chromadorea	Neochromadora
denovo60	gb|AY854210.1|	97,63	phylum:Nematoda	class:Chromadorea	Neochromadora
denovo10	gb|AY854210.1|	95,78	phylum:Nematoda	class:Chromadorea	Neochromadora
denovo193	gb|AY854210.1|	95,51	phylum:Nematoda	class:Chromadorea	Neochromadora
denovo48	gb|AY854210.1|	93,95	phylum:Nematoda	class:Chromadorea	Neochromadora
denovo333	gb|JN968246.1|	94,72	phylum:Nematoda	class:Chromadorea	Neochromadora sp.
denovo207	gb|JN968246.1|	93,14	phylum:Nematoda	class:Chromadorea	Neochromadora sp.
denovo78	gb|JN968246.1|	96,31	phylum:Nematoda	class:Chromadorea	Neochromadora sp.
denovo197	gb|JN968246.1|	92,61	phylum:Nematoda	class:Chromadorea	Neochromadora sp.
denovo154	gb|JN968246.1|	95,78	phylum:Nematoda	class:Chromadorea	Neochromadora sp.
denovo194	gb|JN968246.1|	94,74	phylum:Nematoda	class:Chromadorea	Neochromadora sp.
denovo261	gb|JN968246.1|	93,44	phylum:Nematoda	class:Chromadorea	Neochromadora sp.
denovo25	gb|JN968215.1|	95,25	phylum:Nematoda	class:Chromadorea	Neochromadora sp.
denovo96	gb|JN968215.1|	91,6	phylum:Nematoda	class:Chromadorea	Neochromadora sp.
denovo66	gb|FJ040459.1|	97,6	phylum:Nematoda	class:Chromadorea	Odontophora sp.
denovo139	gb|FJ040459.1|	94,43	phylum:Nematoda	class:Chromadorea	Odontophora sp.
denovo289	gb|AY854196.1|	96,32	phylum:Nematoda	class:Enoplea	Odontophora sp.
denovo300	gb|FJ040499.1|	96,55	phylum:Nematoda	class:Enoplea	Oxystomina sp.
denovo277	gb|FJ040499.1|	95,78	phylum:Nematoda	class:Enoplea	Oxystomina sp.
denovo274	gb|FJ040499.1|	96,55	phylum:Nematoda	class:Enoplea	Oxystomina sp.
denovo57	gb|KJ638035.1|	95,89	phylum:Nematoda	class:Chromadorea	Paracanthonchus sp.
denovo258	gb|KF591743.1|	92,73	phylum:Nematoda	class:Chromadorea	Pomponema sp.
denovo0	gb|JF293023.1|	98,73	phylum:Nemertea	class:Enopla	Prosorhochmus americanus
denovo316	gb|JN968227.1|	90,81	phylum:Nematoda	class:Chromadorea	Punctodora ratzeburgensis
denovo117	gb|JN968228.1|	98,43	phylum:Nematoda	class:Chromadorea	Sabatieria pulchra
denovo19	gb|JN968228.1|	91,95	phylum:Nematoda	class:Chromadorea	Sabatieria pulchra
denovo141	gb|JN968228.1|	97,45	phylum:Nematoda	class:Chromadorea	Sabatieria pulchra
denovo183	gb|JN968221.1|	97,97	phylum:Nematoda	class:Chromadorea	Sabatieria sp.
denovo43	gb|JN968221.1|	92,15	phylum:Nematoda	class:Chromadorea	Sabatieria sp.
denovo101	gb|JN968221.1|	97,38	phylum:Nematoda	class:Chromadorea	Sabatieria sp.
denovo113	gb|JN968221.1|	94,5	phylum:Nematoda	class:Chromadorea	Sabatieria sp.
denovo68	gb|EF591321.1|	95,26	phylum:Nematoda	class:Chromadorea	Setostephanolaimus spartinae
denovo29	gb|JN968264.1|	95,36	phylum:Nematoda	class:Chromadorea	Sphaerolaimus hirsutus
denovo180	gb|JN968239.1|	91,6	phylum:Nematoda	class:Chromadorea	Sphaerolaimus hirsutus
denovo21	gb|JN968216.1|	99,44	phylum:Nematoda	class:Chromadorea	Spirinia parasitifera
denovo31	gb|JN968216.1|	95,24	phylum:Nematoda	class:Chromadorea	Spirinia parasitifera isolate
denovo54	gb|FJ040468.1|	97,87	phylum:Nematoda	class:Chromadorea	Synonchiella sp.
denovo23	gb|AY284683.1|	90,89	phylum:Nematoda	class:Chromadorea	Teratocephalus terrestris
denovo69	gb|JN968231.1|	93,99	phylum:Nematoda	class:Chromadorea	Theristus sp.
denovo89	gb|JN968231.1|	97,14	phylum:Nematoda	class:Chromadorea	Theristus sp.
denovo128	gb|JN968231.1|	95,56	phylum:Nematoda	class:Chromadorea	Theristus sp.
denovo129	gb|AY763130.1|	96,89	phylum:Nematoda	environmental samples	Uncultured nematode
denovo115	gb|AY854198.1|	97,62	phylum:Nematoda	class:Enoplea	Viscosia viscosa
denovo100	gb|AY854198.1|	94,97	phylum:Nematoda	class:Enoplea	Viscosia viscosa
denovo9	gb|KC920423.1|	93,97	phylum:Nematoda	class:Chromadorea	Zygonemella striata
denovo272	gb|KC920423.1|	90,62	phylum:Nematoda	class:Chromadorea	Zygonemella striata

Supplementary Table S1.2- Closest BLAST matches of Operational Taxonomic Units (OTUs) retrieved from Rothera sample sites, assigned to Arthropoda, Annelida and Mollusca up to genus or species levels (Description) using SILVA 1.11 database. Depicted are the public accession numbers (AcNumber), BLAST identity percentage against SILVA (BLAST % ID), Phylum and other Taxa ranking.

OTU#	AcNumber	BLAST % ID	Phylum	Taxa Rank	Description
denovo224	dbj|AB076626.1|	99,74	phylum:Arthropoda	superfamily:Cytheroidea	Howeina sp.
denovo64	dbj|AB076628.1|	95,61	phylum:Arthropoda	superfamily:Cytheroidea	Cytheropteron subuchioi
denovo295	dbj|AB076628.1|	96,38	phylum:Arthropoda	superfamily:Cytheroidea	Cytheropteron subuchioi
denovo36	dbj|AB076644.1|	98,71	phylum:Arthropoda	superfamily:Cytheroidea	Robustaurila salebrosa
denovo200	gb|DQ538499.1|	94,78	phylum:Arthropoda	order:Siphonostomatoida	Kroyeria sp.
denovo322	gb|DQ538499.1|	93,77	phylum:Arthropoda	order:Siphonostomatoida	Kroyeria sp.
denovo238	gb|EU380295.1|	99,22	phylum:Arthropoda	order:Harpacticoida	Dactylopusia sp.
denovo326	gb|AY627016.1|	96,08	phylum:Arthropoda	order:Harpacticoida	Bradya sp.
denovo297	gb|EU380302.1|	93,83	phylum:Arthropoda	order:Harpacticoida	Parastenhelia sp.
denovo103	gb|EU380309.1|	96,87	phylum:Arthropoda	order:Harpacticoida	Itunella muelleri
denovo257	gb|AY627016.1|	97,39	phylum:Arthropoda	order:Harpacticoida	Bradya sp.
denovo53	gb|KC815328.1|	96,86	phylum:Arthropoda	order:Harpacticoida	Amphiascoides atopus
denovo162	gb|AY627015.1|	93,23	phylum:Arthropoda	order:Harpacticoida	Bryocamptus pygmaeus
denovo334	gb|AY627016.1|	98,44	phylum:Arthropoda	order:Harpacticoida	Bradya sp.
denovo233	gb|EU380309.1|	95,3	phylum:Arthropoda	order:Harpacticoida	Itunella muelleri
denovo105	gb|AY627016.1|	97,13	phylum:Arthropoda	order:Harpacticoida	Bradya sp.
denovo201	gb|EU380309.1|	97,65	phylum:Arthropoda	order:Harpacticoida	Itunella muelleri
denovo303	gb|EU380306.1|	98,17	phylum:Arthropoda	order:Harpacticoida	Argestigens sp.
denovo163	gb|EU380285.1|	98,69	phylum:Arthropoda	order:Harpacticoida	Harpacticus sp.
denovo172	gb|AY692343.1|	96,86	phylum:Arthropoda	order:Harpacticoida	Tisbe furcata
denovo273	gb|EU380309.1|	95,05	phylum:Arthropoda	order:Harpacticoida	Itunella muelleri
denovo338	gb|EU380309.1|	95,06	phylum:Arthropoda	order:Harpacticoida	Itunella muelleri
denovo176	gb|EU380309.1|	93,01	phylum:Arthropoda	order:Harpacticoida	Itunella muelleri
denovo265	gb|KC815328.1|	97,38	phylum:Arthropoda	order:Harpacticoida	Amphiascoides atopus
denovo210	gb|AY627016.1|	96,43	phylum:Arthropoda	order:Harpacticoida	Bradya sp.
denovo144	gb|EU380309.1|	95,83	phylum:Arthropoda	order:Harpacticoida	Itunella muelleri
denovo228	gb|EU380306.1|	97,38	phylum:Arthropoda	order:Harpacticoida	Argestigens sp.
denovo119	gb|EU380300.1|	95,05	phylum:Arthropoda	order:Harpacticoida	Paramenophia sp.
denovo234	gb|EU380309.1|	96,87	phylum:Arthropoda	order:Harpacticoida	Itunella muelleri
denovo332	gb|EU380297.1|	95,4	phylum:Arthropoda	order:Harpacticoida	Diarthrodes sp.
denovo324	gb|EU380309.1|	97,14	phylum:Arthropoda	order:Harpacticoida	Itunella muelleri
denovo1	gb|EU380309.1|	93,75	phylum:Arthropoda	order:Harpacticoida	Itunella muelleri
denovo11	gb|EU380309.1|	94,27	phylum:Arthropoda	order:Harpacticoida	Itunella muelleri
denovo132	gb|EU380309.1|	93,99	phylum:Arthropoda	order:Harpacticoida	Itunella muelleri
denovo12	gb|EU380303.1|	98,69	phylum:Arthropoda	order:Harpacticoida	Ameira scotti
denovo77	gb|EU380299.1|	96,08	phylum:Arthropoda	order:Harpacticoida	Sewellia tropica
denovo121	gb|EU380306.1|	96,82	phylum:Arthropoda	order:Harpacticoida	Argestigens sp.
denovo135	gb|EU380295.1|	95,04	phylum:Arthropoda	order:Harpacticoida	Dactylopusia sp.
denovo190	gb|EU380309.1|	96,72	phylum:Arthropoda	order:Harpacticoida	Itunella muelleri
denovo212	gb|EU380309.1|	94,26	phylum:Arthropoda	order:Harpacticoida	Itunella muelleri
denovo226	gb|EU380309.1|	92,72	phylum:Arthropoda	order:Harpacticoida	Itunella muelleri
denovo229	gb|EU380295.1|	95,34	phylum:Arthropoda	order:Harpacticoida	Dactylopusia sp.
denovo291	gb|EU380295.1|	95,48	phylum:Arthropoda	order:Harpacticoida	Dactylopusia sp.
denovo305	gb|EU380297.1|	95,09	phylum:Arthropoda	order:Harpacticoida	Diarthrodes sp.
denovo307	gb|EU380309.1|	95,6	phylum:Arthropoda	order:Harpacticoida	Itunella muelleri
denovo280	gb|AY118078.2|	100	phylum:Arthropoda	order:Calanoida	Ctenocalanus citer
denovo99	gb|FJ372639.1|	93,75	phylum:Arthropoda	infraclass:Paraneoptera	Saldula sp.
denovo2	emb|AJ238061.1|	100	phylum:Arthropoda	genus:Artemia	Artemia franciscana
denovo283	gb|JQ000095.1|	99,22	phylum:Arthropoda	family:Glycyphagidae	Marsupialichus brasiliensis
denovo179	gb|GU902153.1|	100	phylum:Annelida	Clitellata	Grania sp.
denovo206	gb|AF411887.1|	99,74	phylum:Annelida	Clitellata	Heronidrilus gravidus
denovo157	gb|JN936459.1|	99,23	phylum:Annelida	class:Polychaeta	Tharyx sp.
denovo309	gb|AF448150.1|	98,73	phylum:Annelida	class:Polychaeta	Apistobranchus typicus
denovo6	gb|JN852836.1|	100	phylum:Annelida	class:Polychaeta	Neopolynoe paradoxa
denovo329	gb|GU179368.1|	100	phylum:Annelida	class:Polychaeta	Aglaophamus trissophyllus
denovo158	gb|EU418858.1|	98,74	phylum:Annelida	class:Polychaeta	Polycirrus sp.
denovo331	gb|JF509728.1|	96,34	phylum:Annelida	class:Polychaeta	Capitella teleta
denovo182	gb|AY525627.1|	94,85	phylum:Annelida	class:Polychaeta	Eulalia viridis
denovo192	gb|AF508126.1|	96,15	phylum:Annelida	class:Polychaeta	Scoloplos johnstonei
denovo104	gb|DQ153064.1|	99,74	phylum:Annelida	class:Polychaeta	Polygordius jouinae
denovo259	gb|AY532362.1|	92,33	phylum:Annelida	class:Polychaeta	Phylo michaelseni
denovo145	gb|KF511823.1|	99,74	phylum:Annelida	class:Polychaeta	Ophelina sp.
denovo73	gb|KC984696.1|	100	phylum:Mollusca	class:Bivalvia	Yoldia eightsi
denovo127	gb|KC429382.1|	100	phylum:Mollusca	class:Bivalvia	Cyamiomactra laminifera
denovo164	gb|JQ611498.1|	100	phylum:Mollusca	class:Bivalvia	Pecten jacobaeus
denovo111	dbj|AB714767.1|	97,69	phylum:Mollusca	class:Bivalvia	Nipponomontacuta actinariophila
denovo312	gb|KC429372.1|	99,74	phylum:Mollusca	class:Bivalvia	Mysella charcoti
denovo56	gb|KC429331.1|	100	phylum:Mollusca	class:Bivalvia	Mytilus edulis
denovo40	gb|KC984695.1|	95,66	phylum:Mollusca	class:Bivalvia	Neilonella whoii
denovo221	gb|KC429382.1|	97,49	phylum:Mollusca	class:Bivalvia	Cyamiomactra laminifera

Supplementary Table S1.3- Closest BLAST matches of Operational Taxonomic Units (OTUs) retrieved from Rothera sample sites, assigned to Platyhelminthes, up to genus or species levels (Description) using SILVA 1.11 database. Depicted are the public accession numbers (AcNumber), BLAST identity percentage against SILVA (BLAST % ID), Phylum and other Taxa ranking.

OTU#	AcNumber	BLAST % ID	Phylum	Taxa Rank	Description
denovo47	emb|AJ012531.1|	95,84	phylum:Platyhelminthes	order:Macrostomida	Paromalostomum fusculum
denovo230	emb|AJ012531.1|	93,54	phylum:Platyhelminthes	order:Macrostomida	Paromalostomum fusculum
denovo34	emb|AJ012531.1|	93,75	phylum:Platyhelminthes	order:Macrostomida	Paromalostomum fusculum
denovo74	emb|AJ012531.1|	93,51	phylum:Platyhelminthes	order:Macrostomida	Paromalostomum fusculum
denovo262	emb|AJ012531.1|	92,45	phylum:Platyhelminthes	order:Macrostomida	Paromalostomum fusculum
denovo260	emb|AJ012531.1|	93,51	phylum:Platyhelminthes	order:Macrostomida	Paromalostomum fusculum
denovo14	gb|KC869790.1|	94,59	phylum:Platyhelminthes	order:Macrostomida	Macrostomum sp.
denovo79	emb|AJ012531.1|	92,99	phylum:Platyhelminthes	order:Macrostomida	Paromalostomum fusculum
denovo94	emb|AJ012531.1|	93,77	phylum:Platyhelminthes	order:Macrostomida	Paromalostomum fusculum
denovo175	emb|AJ012531.1|	94,06	phylum:Platyhelminthes	order:Macrostomida	Paromalostomum fusculum
denovo243	emb|AJ012531.1|	93,01	phylum:Platyhelminthes	order:Macrostomida	Paromalostomum fusculum
denovo218	gb|KC529506.1|	96,13	phylum:Platyhelminthes	suborder:Dalyellioida	Pogaina sp.
denovo50	gb|KC602396.1|	94,85	phylum:Platyhelminthes	suborder:Kalyptorhynchia	Acrorhynchides robustus
denovo67	gb|KJ887470.1|	95,03	phylum:Platyhelminthes	suborder:Kalyptorhynchia	Uncinorhynchus flavidus v
denovo33	gb|KC529411.1|	96,34	phylum:Platyhelminthes	suborder:Neodalyellida	Proxenetes puccinellicola
denovo16	gb|KC529435.1|	93,93	phylum:Platyhelminthes	suborder:Neodalyellida	Byrsophlebs delamarei
denovo186	gb|AY775738.1|	97,91	phylum:Platyhelminthes	suborder:Kalyptorhynchia	Stradorhynchus sp.
denovo203	gb|AY775741.1|	94,04	phylum:Platyhelminthes	suborder:Kalyptorhynchia	Mesorhynchus terminostylus
denovo340	gb|KJ887440.1|	98,95	phylum:Platyhelminthes	suborder:Kalyptorhynchia	Odontorhynchus aculeatus
denovo7	gb|KJ887470.1|	97,9	phylum:Platyhelminthes	suborder:Kalyptorhynchia	Uncinorhynchus flavidus
denovo17	emb|AJ012507.1|	91,67	phylum:Platyhelminthes	suborder:Kalyptorhynchia	Cheliplana cf. orthocirra
denovo61	gb|KJ887445.1|	94,52	phylum:Platyhelminthes	suborder:Kalyptorhynchia	Opisthocystis goettei
denovo282	gb|KC529506.1|	94,07	phylum:Platyhelminthes	suborder:Dalyellioida	Pogaina sp. 3
denovo98	gb|KC529523.1|	95,63	phylum:Platyhelminthes	suborder:Dalyellioida	Dalyellioida sp.
denovo279	gb|GU936108.1|	93,19	phylum:Platyhelminthes	suborder:Kalyptorhynchia	Schizorhynchidae sp.
denovo239	gb|KJ887448.1|	94,79	phylum:Platyhelminthes	suborder:Kalyptorhynchia	Thylacorhynchus conglobatus
denovo320	gb|KC602396.1|	93,56	phylum:Platyhelminthes	suborder:Kalyptorhynchia	Acrorhynchides robustus
denovo41	gb|KC602396.1|	93,04	phylum:Platyhelminthes	suborder:Kalyptorhynchia	Acrorhynchides robustus
denovo63	gb|AY775746.1|	100	phylum:Platyhelminthes	suborder:Kalyptorhynchia	Schizochilus choriurus
denovo107	gb|KC529518.1|	93,79	phylum:Platyhelminthes	suborder:Dalyellioida	Wahlia macrostylifera
denovo146	gb|KC529521.1|	92,98	phylum:Platyhelminthes	suborder:Typhloplanoida	Austradenopharynx sp.
denovo185	gb|KC529506.1|	96,66	phylum:Platyhelminthes	suborder:Dalyellioida	Pogaina sp.
denovo271	gb|KC869833.1|	92,54	phylum:Platyhelminthes	suborder:Dalyellioida	Baicalellia canadensis
denovo290	gb|KC869833.1|	96,39	phylum:Platyhelminthes	suborder:Dalyellioida	Baicalellia canadensis
denovo313	gb|U70077.1|ARU70	92,23	phylum:Platyhelminthes	order:Proseriata	Archiloa rivularis
denovo268	gb|AY775733.1|	99,74	phylum:Platyhelminthes	order:Proseriata	Cirrifera sopottehlersae
denovo199	gb|AY222124.1|	94,72	phylum:Platyhelminthes	order:Plagiorchiida	Enenterum aureum

Supplementary Table S2: Overview of the Antarctic sampled sites *in silico* statistics for the NGS of 18S rRNA gene region used. Each replicated sampled site had a 8 nucleotide multiplex-identification tag (MID), depth in meters (m), abbreviated description of the sample, post-quality control and chimera checked number of reads and total number of OTUs at the 97% threshold.

Location	MIDTag	Depth (m)	Description	No Reads	QC/ Chimera check reads	Total OTUs
Hangar_1	TCGTCTAC	18	HC.1	1224	970	49
Hangar_2	AGACAGAC	18	HC.2	13007	10399	104
Hangar_3	CTGTTCAC	18	HC.3	4160	3076	117
Rothera Point_1	AGTCAGAG	15	RP.1	402	341	37
Rothera Point_2	TCAGCTCT	15	RP.2	478	376	30
Rothera Point3	ACTCAGAC	15	RP.3	7230	6181	79
Islands_1	CTAGTCCT	13	I.1	19716	16730	157
Islands_2	CAGTTGAC	13	I.2	549	455	54
Islands_3	TAGGTTGC	13	I.3	3617	2924	50
South cove_1	TCTGCTCA	8	SC.1	424	337	21
South cove_2	ATCGTAGC	8	SC.2	4767	4034	51
South cove_3	CATGTGCA	8	SC.3	549	3832	69
